# Hormonal and Transcriptomic Insights into Inflorescence Stalk Elongation in Oil Palm

**DOI:** 10.3390/plants14111715

**Published:** 2025-06-04

**Authors:** Peng Shi, Yin Min Htwe, Dapeng Zhang, Zhiying Li, Qun Yu, Xiangman He, Jing Yang, Yong Wang

**Affiliations:** 1 National Key Laboratory for Tropical Crop Breeding/Sanya Research Institute, Chinese Academy of Tropical Agricultural Sciences, Sanya 572000, China; ship@catas.cn (P.S.); yinminhtwemgk@gmail.com (Y.M.H.); zhangdp@catas.cn (D.Z.); lizhiyingalien@gmail.com (Z.L.); yuqun1998@gmail.com (Q.Y.); 18389739413@163.com (X.H.); 2 Hainan Key Laboratory of Tropical Oil Crops Biology/Coconut Research Institute, Chinese Academy of Tropical Agricultural Sciences, Wenchang 571339, China; 2022316961@stu.ynau.edu.cn

**Keywords:** oil palm, stalk elongation, RNA-seq, hormone

## Abstract

Longer inflorescence stalks in oil palm enhance harvesting efficiency and reduce labor costs. However, the research on this topic is limited. This study aimed to investigate the differences in stalk lengths between male and female inflorescences in Tenera oil palm and to elucidate the underlying hormonal and transcriptomic mechanisms. The stalk lengths from inflorescences associated with the fourth to eighteenth leaf positions of Tenera oil palm trees were measured, and hormone profiling and RNA sequencing (RNA-seq) were conducted in immature (F4 and M5) and mature (F14 and M13) stalks from an individual tree. The male stalks were significantly longer than the female stalks since the thirteenth inflorescences and the differences increased with maturation. The elevated levels of indole-3-acetic acid (IAA) in both immature and mature male stalks suggested auxin’s critical role in promoting stalk elongation. In M13, we identified the upregulated auxin influx carrier *LAX2*, Gibberellic Acid-Stimulated Arabidopsis 6 (*GASA6*), and SMALL AUXIN UP RNA (*SAUR*) genes, indicating enhanced auxin accumulation, signaling, and response. Moreover, the auxin response factor (*ARF11*) was upregulated, linking auxin transport to gene activation for cell elongation. Conversely, in F14, higher levels of abscisic acid (ABA) and the expression of ABA receptor *PYL3* and gibberellin 2-beta-dioxygenase 8 *GA2ox8*, which may inhibit stalk elongation, were identified. The results suggested that *LAX2*-mediated IAA accumulation activates *ARF11* and SAURs, promoting stalk elongation, with *GASA6* possibly acting as a downstream modulator. This study provides insights into the hormonal and genetic regulators of stalk elongation in oil palm and may guide breeding strategies for oil palm varieties with longer stalks of female inflorescences, thereby enhancing harvesting efficiency.

## 1. Introduction

Oil palm (*Elaeis guineensis*) is a crucial crop in the global agricultural industry, known for its high oil yield [1]. One critical factor influencing harvest efficiency and overall productivity is the length of inflorescence stalks, particularly in female plants. Short stalks hinder mechanical harvesting by limiting access to fruit bunches, increasing the risk of fruit damage, and reducing overall yield. In contrast, longer stalks improve harvesting efficiency and reduce labor costs by allowing the easier and safer detachment of fruit bunches—key advantages for large-scale oil palm production systems [2]. Research into the factors influencing stalk length in oil palm is, therefore, of great importance. Understanding these factors could help in developing new oil palm varieties with longer stalks of female inflorescences, thereby improving harvest efficiency and reducing associated costs. Despite its agricultural importance, the regulation of inflorescence stalk elongation in oil palm remains poorly understood. Notably, male inflorescence stalks are generally longer than those of females, providing a useful biological contrast for investigating the factors involved in stalk elongation.

Plant hormones, particularly auxin, play an essential role in cell elongation and organ growth. Auxin promotes cell wall loosening, enabling cell expansion and contributing to cell elongation [3]. In Chinese cabbage, auxin–gibberellin crosstalk regulates stalk elongation [4]. Auxin-responsive genes also play significant roles in various plant species. For instance, in cucumber, the auxin-responsive gene *CsSAUR31* is highly expressed in the roots and male flowers, promoting root and hypocotyl elongation when overexpressed [5]. Similarly, in *Arabidopsis*, the *SAUR63* gene promotes auxin-stimulated organ elongation [6]. Other hormones such as gibberellins, cytokinins, and brassinosteroids also contribute to stalk development through intricate signaling pathways [7]. These findings underscore the importance of plant hormones and their related genes in regulating cell and organ elongation across different plant species.

In addition to hormonal signaling, transcription factors (TFs) play key roles in regulating plant growth [8]. The WRKY transcription factor *WRKY46*, for example, modulates auxin homeostasis in *Arabidopsis* [9], and *OsWRKY78* is associated with stem elongation in rice [10]. Growth-regulating factors (GRFs), including *GRF1* and *GRF3*, influence auxin signaling, thereby regulating plant growth and development [11]. Similarly, MADS-box genes such as *AGL12* have been shown to mediate auxin-dependent cell elongation [12], and GASA family genes are linked to hormone-regulated fiber elongation in cotton [13]. Despite the extensive research identifying several genes and mechanisms involved in organ elongation, related studies on oil palm remain unclear so far.

In this study, we aimed to investigate the molecular mechanisms regulating inflorescence stalk elongation in oil palm. We hypothesized that differences in endogenous hormone levels and gene expression patterns contribute to the distinct stalk lengths observed between female and male inflorescences. To test this hypothesis, we conducted a comparative analysis of female (F) and male (M) stalk lengths from inflorescences at the fourth to eighteenth leaf positions of Tenera oil palm trees. We further analyzed the endogenous hormone content of and performed RNA sequencing (RNA-seq) in immature (F4 and M5) and mature (F14 and M13) inflorescence stalks from an individual tree to identify the key regulators involved in this developmental process.

## 2. Materials and Methods

### 2.1. Sample Preparation

**Figure 1:**
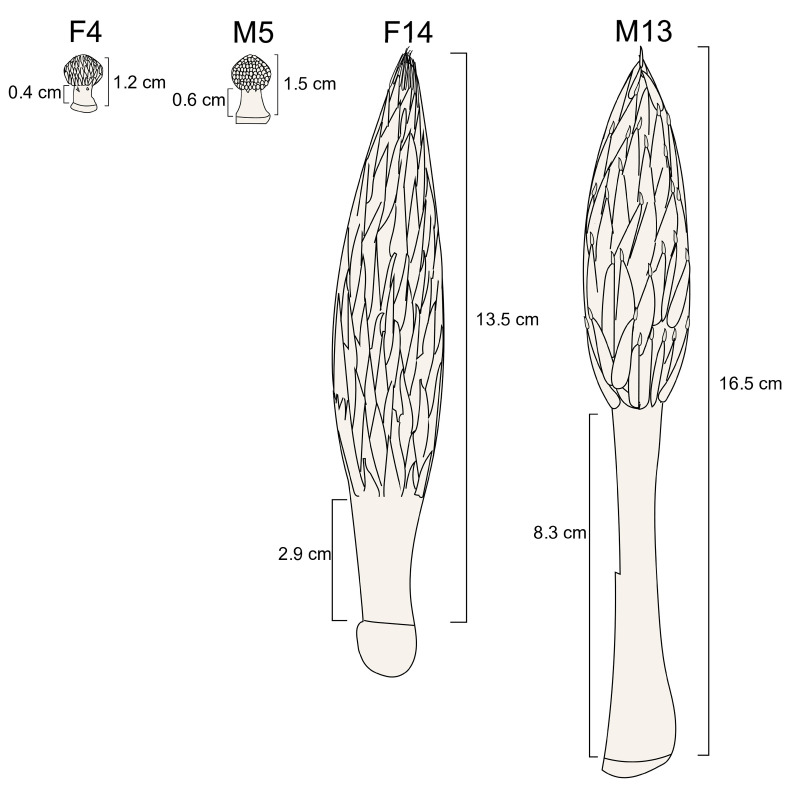
Schematic view of immature female and male (F4 and M5) and mature female and male (F14 and M13) inflorescences.

To analyze the differences in stalk lengths, the stalk lengths of inflorescences from the 4th to 18th leaf positions of male (M) or female (F) Tenera oil palm trees were measured. For RNA sequencing (RNA seq) and endogenous hormone analysis, male inflorescences from the 5th and 13th leaves (M5 and M13) and female inflorescences from the 4th and 14th leaves (F4 and F14) from an individual tree were harvested before bracts split (Figure 1). These tissues were immediately frozen in liquid nitrogen and stored at −80 °C for subsequent analyses.

### 2.2. Determination of Endogenous Hormones

HPLC-grade acetonitrile (ACN) and methanol (MeOH) were purchased from Merck (Darmstadt, Germany). MilliQ water (Millipore, Bradford, PA, USA) was used in all experiments. All of the standards were purchased from Olchemim Ltd. (Olomouc, Czech Republic) and Sigma (St. Louis, MO, USA). Acetic acid and formic acid were bought from Sinopharm Chemical Reagent (Shanghai, China). The stock solutions of standards were prepared at the concentration of 1 mg/mL in MeOH and stored at −20 °C. The stock solutions were diluted with MeOH to give working solutions before analysis.

Fresh plant materials were harvested, weighed, immediately frozen in liquid nitrogen, and stored at −80 °C until needed. For hormone extraction, 50 mg of fresh plant tissue was ground into a fine powder in liquid nitrogen and extracted with 1 mL methanol/water/formic acid mixture (15:4:1, *V/V/V*). The combined extracts were evaporated to dryness under nitrogen gas stream, reconstituted in 100 μL of 80% methanol (*V/V*), and filtered through a 0.22 μm filter for subsequent LC-MS analysis.

The extracts were analyzed using an LC-ESI-MS/MS system (UHPLC, ExionLC^TM^ AD, SCIEX, Framingham, MA, USA; MS, Applied Biosystems 6500 Triple Quadrupole, SCIEX, Framingham, MA, USA). The analytical conditions were as follows: HPLC column, Waters ACQUITY UPLC HSS T3 C18 (Waters Corporation, Milford, MA, USA) (100 mm × 2.1 mm i.d., 1.8 µm); solvent system, water with 0.04% acetic acid (A), acetonitrile with 0.04% acetic acid (B); gradient program, started at 5% B (0–1 min), increased to 95% B (1–8 min), 95% B (8–9 min), and finally ramped back to 5% B (9.1–12 min); flow rate, 0.35 mL/min; temperature, 40 °C; and injection volume, 2 μL.

Mass spectrometric detection was performed using an AB 6500+ QTRAP^®^ LC-MS/MS System (SCIEX, Framingham, MA, USA) equipped with an ESI Turbo Ion-Spray interface, operating in both positive and negative ion modes, and controlled by Analyst 1.6 software (AB Sciex, Framingham, MA, USA).

The ESI source operation parameters were as follows: turbo spray ion source, source temperature of 550 °C, ion spray voltage (IS) of 5500 V (positive mode) and −4500 V (negative mode), and curtain gas (CUR) set at 35.0 psi. Specific multiple reaction monitoring (MRM) transitions were optimized for declustering potential (DP) and collision energy (CE) for each phytohormone. Phytohormone contents were quantified by MetWare (http://www.metware.cn/) accessed on 23 March 2023 using the AB Sciex QTRAP 6500 LC-MS/MS platform.

This hormone extraction protocol and LC-MS/MS analytical method was performed following protocols described by Pan et al. (2010) [14].

### 2.3. RNA Extraction, Library Construction, and Sequencing

Total RNA was extracted using Trizol reagent kit (Invitrogen, Carlsbad, CA, USA) according to manufacturer’s instruction. RNA quality was assessed on an Agilent 2100 Bioanalyzer (Agilent Technologies, Palo Alto, CA, USA) and checked using RNase-free agarose gel electrophoresis.

After total RNA was extracted, eukaryotic mRNA was enriched by Oligo(dT) beads, while prokaryotic mRNA was enriched by removing rRNA using the Ribo-Zero^TM^ Magnetic Kit (Epicentre, Madison, WI, USA). Then, the enriched mRNA was fragmented into short fragments using fragmentation buffer and reverse transcripted into cDNA with random primers. Second-strand cDNA was synthesized by DNA polymerase I, RNase H, dNTP, and buffer. Then, the cDNA fragments were purified with QiaQuick PCR extraction kit (Qiagen, Venlo, The Netherlands), end repaired, poly(A) added, and ligated to Illumina sequencing adapters. The ligation products were size selected by agarose gel electrophoresis, PCR amplified, and sequenced using Illumina HiSeq2500 by Gene Denovo Biotechnology Co. (Guangzhou, China).

After sequencing, reads were further filtered by fastp [15] (version 0.18.0) with parameters (1) removing reads containing adapters; (2) removing reads containing more than 10% of unknown nucleotides (N); (3) removing low-quality reads containing more than 50% of low-quality (Q-value ≤ 20) bases. Short reads alignment tool Bowtie2 [16] (version 2.2.8) was used for mapping reads to a ribosome RNA (rRNA) database. The rRNA mapped reads were then removed. The remaining clean reads were further used in assembly and gene abundance calculation. An index of the reference genome was built, and paired-end clean reads were mapped to reference genome using HISAT2. 2.4 [17] with “-rna-strandness RF” and other parameters set as a default.

### 2.4. Differentially Expressed Genes (DEGs) Analysis

Gene expression levels were quantified using FPKM (fragments per kilobase of transcript per million mapped reads), which adjusts for both sequencing depth and gene length. Although FPKM was used for descriptive analysis and expression profiling, differential gene expression was identified using raw read counts analyzed by DESeq2 [18] and edgeR [19]. These tools apply internal normalization methods and estimate replicate-level dispersion to account for biological variability. To correct for multiple hypothesis testing, the Benjamini–Hochberg False Discovery Rate (FDR) method [20] was applied. The genes/transcripts with a parameter of false discovery rate (FDR) < 0.05 and absolute fold change ≥ 2 were considered differentially expressed genes/transcripts. Then, gene ontology (GO) classification and Kyoto Encyclopedia of Genes and Genomes (KEGG) pathway enrichment of DEGs were analyzed to evaluate their biological functions. The calculated p-value went through FDR correction, taking FDR ≤ 0.05 as a threshold. GO terms and pathways meeting this condition were defined as significantly enriched GO terms and pathways in DEGs. All analyses were conducted using three biological replicates per group.

### 2.5. Data Analysis and Visualization

Inflorescence lengths, stalk lengths, and endogenous hormone contents were analyzed using Python (version 3.10.9) by Anaconda in Jupyter Notebook (version 6.5.2). All statistical analyses were based on three independent biological replicates. Two-tailed independent *t*-tests were performed to assess differences in stalk lengths and hormone levels between female and male samples. Based on the *p*-values, significance levels were assigned using the following criteria: *p* < 0.001 (***), *p* < 0.01 (**), *p* < 0.05 (*), and *p* ≥ 0.05 (ns). Gene expression data were analyzed from FPKM values of three replicates. Heatmaps, circular dendrograms, and lollipop charts for KEGG pathway analyses were generated using R [21] (version 4.1.0) and Rstudio [22] (version 1.4.1717).

## 3. Results

### 3.1. Differences in Stalk Lengths Between Female and Male Inflorescences

**Figure 2:**
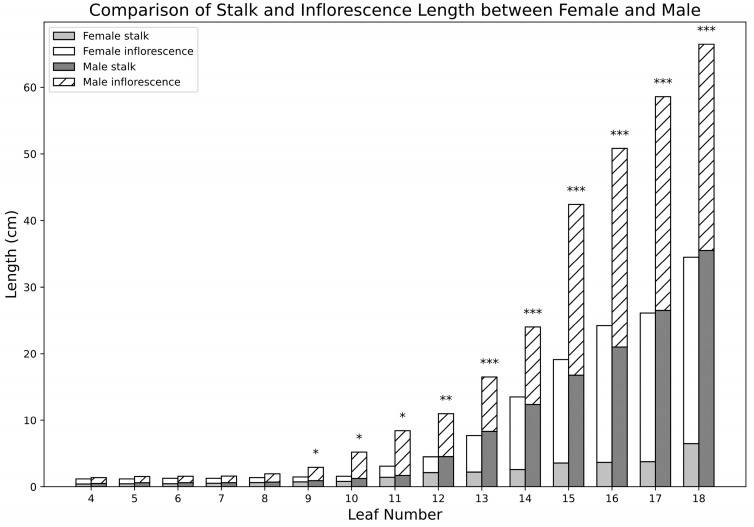
Comparison of female and male inflorescences and stalk lengths from the 4th to the 18th leaves. The bar graphs represent the mean lengths (in cm) of inflorescences and stalks based on three independent biological replicates (*n* = 3). Significant differences between female and male stalk lengths are indicated by asterisks (* *p* < 0.05, ** *p* < 0.01, *** *p* < 0.001).

A comparative analysis of stalk lengths from female (F) and male (M) inflorescences at the fourth to eighteenth leaf positions of Tenera oil palm trees was performed. It is evident that male inflorescences and stalks were consistently longer than those of females across all leaf numbers. The differences in stalk lengths were significant starting from the ninth leaf (*p* < 0.05) and continued to show significant differences throughout the observed leaf numbers, with the most substantial differences (*p* < 0.001) occurring in leaves 13, 14, 15, 16, 17, and 18 (Figure 2).

### 3.2. Quantification of Endogenous Hormones

**Figure 3:**
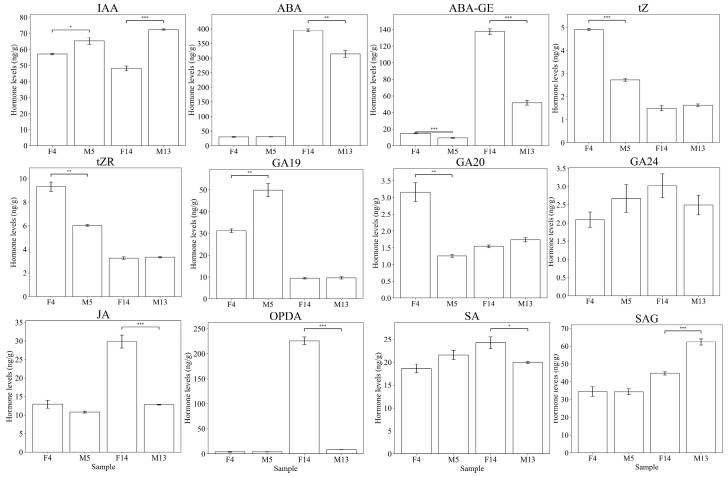
Endogenous hormone levels in immature (F4 and M5) and mature (F14 and M13) inflorescence stalks of oil palm. Each bar represents the mean ± standard error of three biological replicates. Significant differences between groups are indicated by asterisks: * *p* < 0.05, ** *p* < 0.01, *** *p* < 0.001.

To elucidate the hormonal dynamics in oil palm inflorescence stalks, the levels of various endogenous hormones were quantified using LC-MS/MS. The hormones analyzed included indole-3-acetic acid (IAA), abscisic acid (abscisic acid: ABA; *ABA–glucose ester*: ABA–GE), cytokinins (trans-zeatin: tZ; trans-zeatin riboside: tZR), gibberellins (GA19, GA20, and GA24), jasmonates (jasmonic acid: JA, oxo-phytodienoic acid: OPDA), and salicylic acid (salicylic acid: SA; salicylic acid glucose ester: SAG). The hormone levels were quantified and expressed as nanograms per gram of fresh weight (ng/g FW). The hormonal profiles were assessed in both immature (F4) and mature (F14) female inflorescence stalks, as well as immature (M5) and mature (M13) male inflorescence stalks (Figure 3).

The results showed that IAA levels were significantly higher in both immature (*p* < 0.05) and mature (*p* < 0.001) male stalks compared with those of female stalks. Abscisic Acid (ABA) levels were significantly higher in F14 compared with M13 (*p* < 0.01). Additionally, ABA–GE levels were significantly elevated in female stalks (F4 and F14) compared with male stalks (M5 and M13) (*p* < 0.001). For cytokinins (tZ, tZR), F4 exhibited significantly higher levels than M5 (*p* < 0.001). A similar trend was observed for tZR, where F4 showed higher hormone levels compared with M5 (*p* < 0.01). The F14 and M13 samples had comparable tZ and tZR levels. GA19 levels were notably higher in M5 compared with F4 (*p* < 0.01), while mature stalks showed no significant difference. GA20 levels were significantly elevated in F4 compared with M5 (*p* < 0.01), whereas no significant differences were detected in mature stalks. GA24 levels did not exhibit significant variation across all the samples. Jasmonates (JA, OPDA) levels were significantly higher in F14 than in M13 (*p* < 0.001). The F4 and M5 samples did not display significant differences in JA and OPDA levels. Salicylic acid (SA) levels were significantly higher in F14 compared with M13 (*p* < 0.05). On the other hand, SAG levels were significantly elevated in M13 compared with F14 (*p* < 0.001). The SA and SAG levels showed no significant differences between F4 and M5.

Overall, the analysis of endogenous hormone levels revealed that the IAA levels were significantly higher in male stalks compared with female stalks, both in mature and immature stages, while ABA levels were significantly higher in mature female stalks (Figure 3).

### 3.3. Transcriptomic Analysis

To investigate the molecular mechanisms underlying stalk elongation, we selected inflorescences from the fourth and fourteenth leaf positions in female palms (F4 and F14) and the fifth and thirteenth leaf positions in male palms (M5 and M13) for RNA sequencing (RNA-seq). Among these samples, M13 exhibited the longest stalk length (8.3 cm) (Figure 2). Twelve RNA-seq libraries were generated from these samples, with three biological replicates each. Each library produced over 36 million high-quality clean reads, with the percentage of high-quality reads exceeding 99% among the raw reads (Table S1).

**Figure 4:**
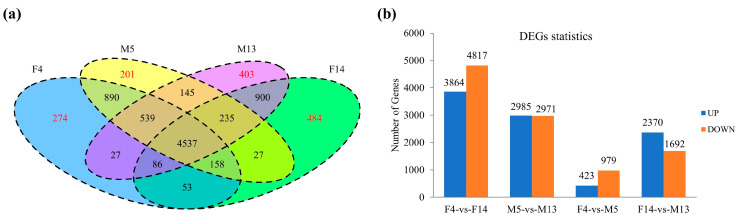
Transcriptomic analysis of stalk length regulation. (**a**) Venn diagram of gene expression. Venn diagram illustrating the total number of genes identified across the four samples (F4, F14, M5, and M13). The red text highlights the number of genes highly expressed in each sample with FPKM ≥ 20. (**b**) Differentially expressed genes (DEGs) statistics. Bar graphs show the number of upregulated and downregulated DEGs in pairwise comparisons: F4 vs. F14, M5 vs. M13, F4 vs. M5, and F14 vs. M13.

Gene expression levels were determined using the FPKM method, identifying a total of 27,913 genes. To focus on highly expressed genes, we filtered for those with an FPKM ≥ 20. The Venn diagram in Figure 4a illustrates the overlap and highly expressed genes across four samples: F4, M5, M13, and F14. In total, 274 genes were highly expressed in F4, 201 in M5, 403 in M13, and 484 in F14, with FPKM ≥ 20 (Figure 4a). Detailed gene expression data and related information for all samples are provided in Table S2.

A total of 8681 differentially expressed genes (DEGs) in F4 vs. F14; 5956 DEGs in M5 vs. M13; 1402 DEGs in F4 vs. M5, and 4062 DEGs in F14 vs. M13 were identified. Among these DEGs, 3864 were upregulated and 4817 were downregulated in F4 vs. F14; 2985 were upregulated and 2971 were downregulated in M5 vs. M13; 423 were upregulated and 979 were downregulated in F4 vs. M5; and 2370 were upregulated and 1692 DEGs were downregulated in F14 vs. M13 (Figure 4b; Table S3). The higher number of DEGs in the female-to-female (F4 vs. F14) and male-to-male (M5 vs. M13) comparisons likely reflects distinct developmental stage-specific gene regulation. In these comparisons, the differences in gene expression are primarily driven by the developmental transitions within the same sex. For instance, as female and male inflorescences progress from stage F4 to F14 and M5 to M13, there are substantial changes in gene expression associated with various physiological and morphological processes, such as cell division, and differentiation.

### 3.4. Expression Analysis of DEGs

In our study, the length of mature male stalks is significantly greater (*p* < 0.001) starting from M13 and more notable compared with those of female stalks, in contrast to immature stalks (Figure 2). This significant difference suggests that the genes responsible for regulating stalk length are likely to be identified in mature stalks. Therefore, the DEGs analysis focused on mature stalk samples, specifically comparing female (F14) and male (M13) samples (F14 vs. M13).

**Figure 5:**
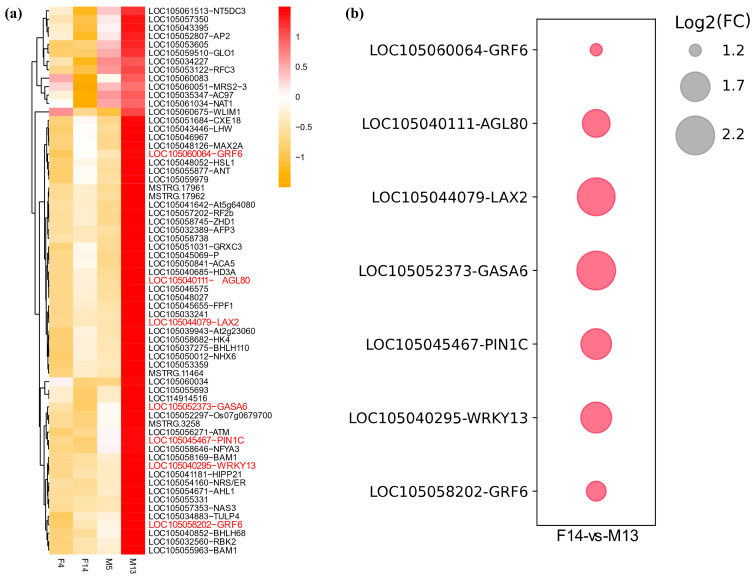
Expression analysis of DEGs in M13. (**a**) Heatmap showing the expression levels of 65 DEGs highly expressed with FPKM ≥ 20 in M13. The color scale represents the expression levels, with red indicating higher expression and yellow indicating lower expression. Key DEGs related to auxin transport, growth regulation, and transcription factors (TFs) are highlighted in red text. (**b**) Bubble plot illustrating the log2 fold change (FC) of selected DEGs in M13 compared with F14. The size of the bubbles corresponds to the magnitude of the log2 FC, with larger bubbles indicating higher log2FC. Gene expression values are derived from three independent biological replicates.

Among the 403 genes highly expressed with FPKM ≥ 20 in M13 (Figure 4a), 65 were differentially expressed in F14 vs. M13 (Figure 5a; Table S4). Among these 65 DEGs, we identified auxin transport genes (*LAX2* and *PIN1C*), Gibberellic Acid-Stimulated Arabidopsis 6 (*GASA6*), and transcription factors such as *GRF6*, *WRKY13*, and agamous-like MADS-box protein *AGL80* (Figure 5), which are reported to play important roles in the promotion of plant growth and development.

**Figure 6:**
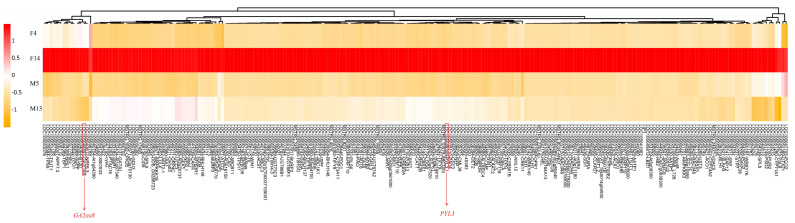
Expression analysis of differentially expressed genes (DEGs) in F14. Heatmap illustrating the expression levels of 226 DEGs highly expressed with FPKM ≥ 20 in F14. The color scale represents the expression levels, with red indicating higher expression and yellow indicating lower expression. DEGs potentially related to short stalks are highlighted in red text. Gene expression values are derived from three independent biological replicates.

Among the 484 genes highly expressed with FPKM ≥ 20 in F14, 226 were differentially expressed in F14 vs. M13. Notably, among these DEGs, we identified the involvement of the abscisic acid receptor *PYL3* and gibberellin 2-beta-dioxygenase 8 *GA2ox8* (Figure 6; Table S5). Previous studies have reported that abscisic acid inhibits hypocotyl elongation [23] and that the overexpression of *AtGA2ox7* and *AtGA2ox8* caused a dwarf phenotype in tobacco [24]. This suggests that *PYL3* and *GA2ox8* in F14 may play key roles in inhibiting stalk elongation in oil palm.

### 3.5. Kyoto Encyclopedia of Genes and Genomes (KEGG) Analysis

**Figure 7:**
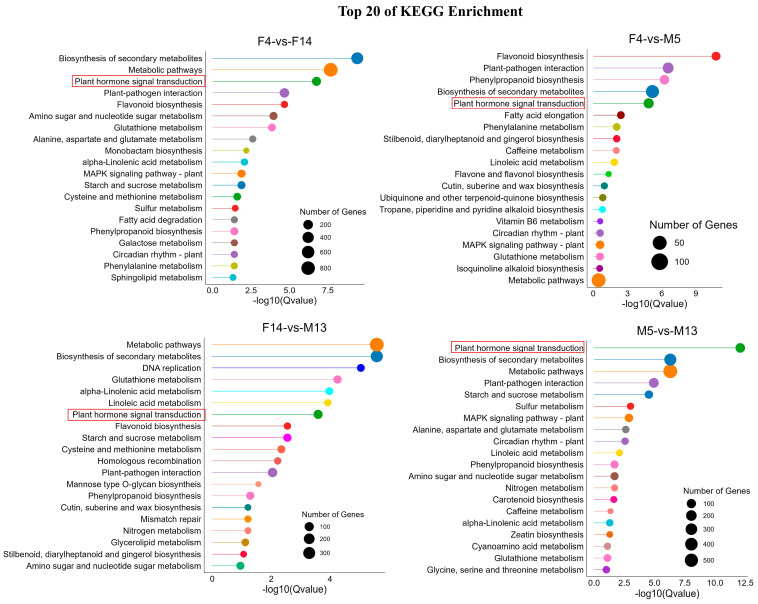
Top 20 Kyoto Encyclopedia of Genes and Genomes (KEGG) pathway enrichments of DEGs between groups. The size of the dots represents the number of genes involved.

The KEGG pathway enrichment analysis for the DEGs in various comparisons highlighted several key pathways (Table S6). Notably, the “plant hormone signal transduction” pathway was enriched across all comparisons, indicating its significant role in regulating stalk development (Figure 7). This finding is consistent with previous reports suggesting that phytohormone signal transduction is involved in stalk developmental processes [4].

**Figure 8:**
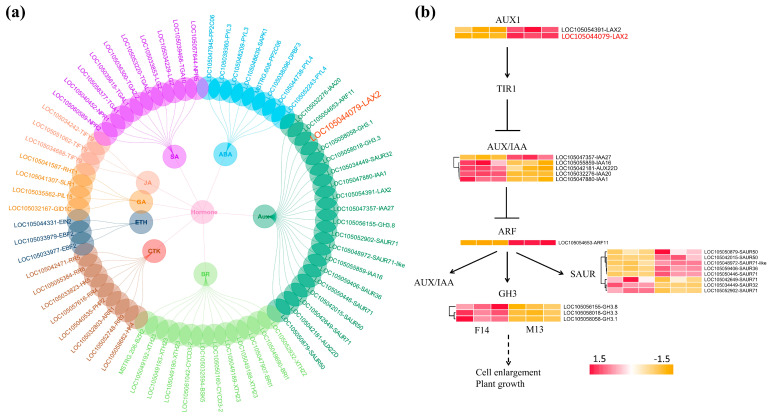
Analysis of plant hormone signal transduction pathways in F14 vs. M13. (**a**) A network diagram depicting the genes involved in various plant hormone signal transduction pathways, including auxin (AUX), cytokinin (CTK), gibberellin (GA), ethylene (ETH), brassinosteroid (BR), jasmonic acid (JA), salicylic acid (SA), and abscisic acid (ABA). The *LAX2* in the auxin signaling pathway, which was highly expressed in M13, is highlighted in red text. (**b**) Detailed illustration of the auxin signaling pathway. The heatmaps show the expression levels of auxin-related genes in F14 and M13, with notable upregulation of *LAX2* in M13. The color scale represents the log2 (FC) in gene expression levels, with red indicating higher expression and yellow indicating lower expression. Gene expression values are derived from three independent biological replicates.

Given the crucial roles of plant hormones in various aspects of plant growth and development [25,26,27] and the significant enrichment of the plant hormone signal transduction pathway in F14 vs. M13, we further analyzed this pathway to understand their roles in regulating stalk lengths between female (F14) and male (M13) samples. A total of 67 DEGs were identified, of which 36 were upregulated and 31 were downregulated (Table S7). The genes involved in the auxin (Aux), cytokinin (CTK), gibberellin (GA), ethylene (ETH), brassinosteroid (BR), jasmonic acid (JA), salicylic acid (SA), and abscisic acid (ABA) signaling pathways were differentially expressed (Figure 8a), suggesting their potential roles in contributing to stalk length development. Among these genes, the auxin influx carrier *LAX2* in the auxin signaling pathway, which was highly expressed in M13 (Figure 5), exhibited upregulated expression. This suggests that auxin plays a critical role in stalk elongation. This finding is consistent with our results from endogenous hormone quantification (Figure 3). Thus, we performed a detailed analysis of the expressions of DEGs in the auxin signaling pathway. A total of 19 DEGs were expressed in the auxin signaling pathway. Notably, the *LAX2*, auxin response factor 11 (*ARF11*), auxin-responsive protein *SAUR50, SAUR71*, and *SAUR36* genes were upregulated in this pathway. In contrast, most of the negative regulators of auxin response (AUX/IAA) were downregulated in M13 (Figure 8b).

## 4. Discussion

Inflorescences with longer stalks are easier to access and handle during oil palm harvesting. This can reduce labor costs and improve harvesting efficiency, as workers or mechanical harvesters can more easily reach and cut the fruit bunches without damaging the plant or the fruits. Emphasizing the advantages of longer stalks can help oil palm cultivators simplify harvesting and improve yields. However, studies in this field are still limited. To address this gap, we analyzed the stalk lengths of male and female inflorescences from the fourth to the eighteenth leaves and found that male inflorescence stalks are significantly longer than female stalks (Figure 2).

As plant hormones regulate various aspects of plant growth and development, we further quantified the endogenous hormones in immature female (F4) and male (M5) inflorescence stalks, as well as mature female (F14) and male (M13) inflorescence stalks (Figure 3). Hormone profiling revealed distinct dynamics between female and male inflorescence stalks that help explain differences in their lengths. The elevated levels of indole-3-acetic acid (IAA) in both immature and mature male inflorescence stalks (M5 and M13) suggest that auxin plays a crucial role in promoting the elongation of male stalks. Auxin is known to facilitate cell elongation and division [3], and its higher concentration in male stalks aligns with the observed longer stalk lengths in males. This agrees with earlier studies showing auxin’s key role in stalk development [4]. Similar to the inhibitory effects of abscisic acid (ABA) on hypocotyl elongation [23], the significantly higher levels of ABA in F14 compared with M13 suggested that ABA may also inhibit stalk elongation in oil palm. In contrast, female inflorescence stalks—particularly the mature F14 stage—exhibited significantly higher levels of abscisic acid (ABA) and its conjugated form ABA–GE, as well as an elevated expression of the abscisic acid receptor *PYL3* and gibberellin 2-beta-dioxygenase *GA2ox8* (Figure 6). These results suggest the presence of an antagonistic hormonal environment in female stalks that may inhibit elongation. It is well documented that ABA suppresses cell expansion by promoting growth-inhibitory pathways, including the stabilization of DELLA proteins and the repression of gibberellin biosynthesis [23]. Meanwhile, *GA2ox8* is a negative regulator of gibberellin activity through the deactivation of bioactive GAs, and its overexpression has been associated with dwarf phenotypes in several plant species [24].

This pattern of enriched ABA and *GA2ox8* expression in female tissues, relative to the elevated auxin levels and SAUR gene activity in male tissues, highlights a fundamental divergence in the regulatory network controlling stalk elongation between female and male tissues. It is possible that female stalks maintain higher levels of ABA and GA-inactivating enzymes to restrict elongation as a developmentally programmed mechanism, possibly to prioritize resource allocation to fruit development. In contrast, male stalks may rely on active auxin signaling and transport to promote elongation, enhancing pollen dispersal potential.

To investigate the possible molecular mechanisms regulating stalk length, we performed RNA sequencing (RNA-seq) analysis on F4, F14, M5, and M13. Among the samples analyzed, M13 had the longest stalk length of 8.3 cm (Figure 1). We identified 403 genes highly expressed with FPKM ≥ 20 in M13, of which 65 were differentially expressed in F14 vs. M13. Among these differentially expressed genes (DEGs) were genes previously reported to play important roles in plant growth and development, including auxin transporters *LAX* and *PIN* [28,29,30], GASA [31,32], and transcription factors (TFs) such as *WRKY* [33], *GRF* [34,35], and *AGL80* [36].

Similarly, RNA-seq analysis in flowering Chinese cabbage revealed the hormone-mediated regulation of stalk development [7]. Consistent with these findings, our analysis revealed a notable enrichment of the plant hormone signal transduction pathway across all comparisons (Figure 7), underscoring its significant role in regulating stalk development. Auxin signaling is known to play a critical role in cell elongation and division [3], which are essential for stalk elongation. In flowering Chinese cabbage, exogenous IAA may regulate stalk elongation by influencing the genes associated with auxin transportation pathways [4]. In *Arabidopsis*, *SAUR36* is reported to be a promoter of organ elongation [5]. In our study, the upregulation of *LAX2*, *ARF11*, and several *SAUR* genes in M13 suggests enhanced auxin transport and response, potentially leading to cell elongation, and subsequently stalk elongation (Figure 8b). The upregulation of *ARF11* and SAURs in M13 may facilitate cell elongation through the downstream activation of genes involved in cell wall loosening. These gene expression results align with the observed hormone profiles (Figure 3), further supporting the role of auxin in promoting stalk elongation.

Among the genes highly expressed in F14, we identified ABA receptor *PYL3* and gibberellin 2-beta-dioxygenase 8 *GA2ox8* (Figure 6). As noted earlier, these genes are likely contributors to stalk elongation repression in female tissues, supported by the literature showing that ABA suppresses hypocotyl elongation [23], and that *GA2ox8* overexpression causes dwarfism through gibberellin deactivation [24]. Thus, the coordinated upregulation of both ABA-related and GA-deactivating genes suggests a hormonally repressive environment that limits elongation in female stalks.

Transcription factors (TFs) play a crucial role in regulating the complex signaling pathways that control plant development [8]. In the current study, we identified TFs highly expressed with FPKM ≥ 20 in M13, including *WRKY13*, *GRF6*, and *AGL80*. These TFs are known to participate in various signaling pathways, particularly those involving plant hormones, which are crucial for plant growth and development. The *WRKY* TFs, for instance, are integral to the signaling pathways of several plant hormones [37,38]. Previous research has demonstrated that *WRKY46* in *Arabidopsis* is involved in regulating auxin homeostasis [9], while *OsWRKY78* in rice regulates stem elongation and cell length [10]. These findings suggest that *WRKY* TFs in oil palm could similarly influence stalk length by modulating hormone levels and signaling pathways. Growth-regulating factors (GRFs) have diverse roles in plant growth and development by interacting with plant hormones [8,11]. GRFs, such as *GRF1* and *GRF3*, are known to regulate auxin levels and responses [11], while *OsGRF1* has been implicated in gibberellin-induced stem elongation [35]. Their studies along with our findings suggest that *GRF6* in oil palm could contribute to stalk length elongation in male inflorescences by regulating auxin accumulation. The agamous-like MADS-box protein *AGL80* also appears to play a significant role. An AGAMOUS-related MADS-box gene, such as *XAL1* (*AGL12*), may act as a mediator of auxin signaling and is implicated in cell elongation [12]. Additionally, the MADS-box gene, *XAL2*, modulates auxin transport by regulating the expression of auxin efflux transporters, such as *PIN* [30]. Therefore, *AGL80* may influence stalk length through its effects on auxin signaling and transport. In addition, *GASA* proteins are reported to be involved in the signal transmission of plant hormones [31]. In cotton, IAA significantly upregulates *GhGASA10–1*, highlighting IAA’s crucial role in fiber cell elongation through its interaction with *GASA* [13]. This suggests that similar mechanisms could be at play in oil palm, where *GASA* may influence stalk elongation through their interaction with IAA.

These insights provide a potential foundation for incorporating molecular tools into oil palm breeding programs. The identified genes, particularly those involved in auxin transport and response (e.g., *LAX2, ARF11*, and SAURs), and gibberellin regulation (*GASA6*) could serve as candidate markers for marker-assisted selection (MAS). For instance, screening breeding populations for favorable alleles or expression profiles of these genes could help identify individuals predisposed to longer stalk phenotypes. Moreover, the hormonal regulatory patterns uncovered here open possibilities for hormone-based treatments or biotechnological interventions, such as the use of CRISPR/Cas9 to manipulate key regulators like *GA2ox8* (a GA-deactivating enzyme) or *PYL3* (an ABA receptor) to relieve growth suppression in female stalks. While further functional validation and field studies are required, these candidate genes represent promising targets for improving harvestability traits in oil palm—a key objective in modern breeding efforts aiming to increase efficiency and reduce labor costs.

One limitation of this study is that the RNA-seq and hormone quantification data were derived from a single individual tree. While this controlled design minimizes confounding variation from genetic and environmental factors, it limits the broader generalizability of the findings. Due to the constraints in sample availability and resources, further validation using independent biological material was not feasible. As such, the results should be interpreted as a proof of concept, laying the groundwork for future studies that expand the validation across multiple individuals and environments.

To summarize these findings, we provide an overview of the key regulatory genes, their expression patterns, hormone pathway associations, and inferred roles in stalk elongation (Table 1).

## 5. Conclusions

**Figure 9:**
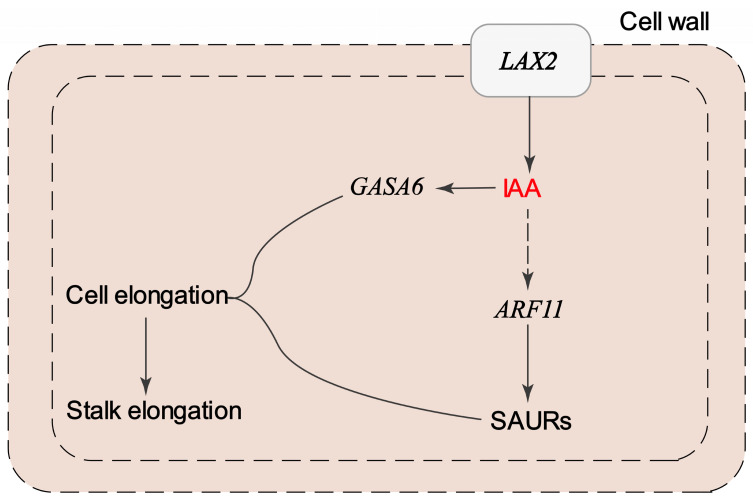
Proposed mechanism regulating stalk elongation. IAA is transported through the cell wall via the influx carrier *LAX2*. Inside the cell, IAA influences the expression of *ARF11*, which subsequently regulates *SAUR* genes. The upregulated expression of SAURs leads to cell elongation, contributing to stalk elongation. Additionally, *GASA6* interacts with this pathway, coordinating the stalk elongation process. Dotted lines in auxin signaling pathway mean there is more than one step during the process.

In summary, the auxin transport and accumulation within a cell are mediated by *LAX2*, leading to the activation of downstream genes such as *ARF11* and SAURs, thereby promoting stalk elongation. Additionally, *GASA6*, likely regulated by IAA, may coordinate this elongation process (Figure 9). Notably, no studies in palms have directly linked *LAX2* or *GASA6* to stalk elongation, highlighting the novelty of our findings. These findings provide valuable insights into the potential molecular mechanisms underlying stalk elongation in oil palm and offer a foundation for breeding strategies aimed at developing varieties with longer stalks of female inflorescences for improved mechanical harvesting. Genes such as *LAX2* and *GASA6* could be further evaluated as functional targets for gene editing or marker development, facilitating the selection or engineering of elite oil palm lines with enhanced harvestability. Future validation studies, such as the overexpression or CRISPR/Cas9-mediated knockout of *LAX2* and *GASA6*, will be essential to functionally confirm their roles in stalk elongation and to further refine their application in oil palm improvement programs.

## Figures and Tables

**Table 1:** Summary of key regulatory genes involved in inflorescence stalk elongation.

Gene	Expression Pattern	Hormone Pathway	Inferred Function
*LAX2*	Upregulated in M13	Auxin influx carrier	Promotes IAA transport into cells; contributes to auxin accumulation and signaling in elongating stalks
*ARF11*	Upregulated in M13	Auxin response factor	Activates downstream auxin-responsive genes involved in cell elongation
SAURs	Multiple members upregulated in M13	Auxin-responsive genes	Promote cell wall loosening and elongation; direct targets of ARFs
*GASA6*	Upregulated in M13	Gibberellin associated	May act downstream of auxin–gibberellin crosstalk to promote stalk elongation
*PYL3*	Upregulated in F14	ABA receptor	Suggests a role for ABA signaling modulation in stalk development
*GA2ox8*	Upregulated in F14	Gibberellin metabolism	Inactivates bioactive gibberellins; likely contributes to growth suppression and shorter stalk length in female inflorescences

## Data Availability

The authors declare that all other data supporting the findings of this study are available within the manuscript and its Supplementary Files. The RNA-seq data for this study can be found in the National Genomics Data Center-Genome Sequence Archive (GSA accession: CRA023492) (https://ngdc.cncb.ac.cn/gsa/browse/CRA023492, accessed on 1 June 2025).

## Supplementary Materials

The following supporting information can be downloaded at: https://www.mdpi.com/article/10.3390/plants14111715/s1, Table S1. Quality assessment of reads. Table S2. The quantification of gene expression. Table S3. Analysis of Differentially expressed genes (DEGs). Table S4. Highly expressed DEGs (FPKM ≥ 20) in M13. Table S5. Highly expressed DEGs (FPKM ≥ 20) in F14. Table S6. Analysis of Kyoto Encyclopedia of Genes and Genomes (KEGG) pathways enrichment. Table S7. Plant hormone signal transduction pathway of F14 vs. M13.

## Abbreviations

M, male; F, female; IAA, indole-3-acetic acid; RNA-seq, RNA sequencing; DEGs, differentially expressed genes; FPKM, fragments per kilobase of transcript per million mapped reads; ABA, abscisic acid; ABA–GE, ABA–glucose ester; tZ, trans-zeatin; tZR, trans-zeatin riboside; JA, jasmonic acid; OPDA, oxo-phytodienoic acid; SA, salicylic acid; SAG, salicylic acid glucose ester; Aux, auxin; ARF, auxin response factor; SAUR, SMALL AUXIN UP RNA; CTK, cytokinin; GA, gibberellin; ETH, ethylene; BR, brassinosteroid; GASA6, Gibberellic Acid-Stimulated Arabidopsis 6; KEGG, Kyoto Encyclopedia of Genes and Genomes; TF, transcription factors (TFs); and GRFs, growth-regulating factors.
